# Activation of Hif1α by the Prolylhydroxylase Inhibitor Dimethyoxalyglycine Decreases Radiosensitivity

**DOI:** 10.1371/journal.pone.0026064

**Published:** 2011-10-07

**Authors:** Marina K. Ayrapetov, Chang Xu, Yingli Sun, Kaya Zhu, Kalindi Parmar, Alan D. D'Andrea, Brendan D. Price

**Affiliations:** 1 Division of Genomic Stability and DNA Repair, Department of Radiation Oncology, Dana-Farber Cancer Institutes, Harvard Medical School, Boston, Massachusetts, United States of America; 2 Disease Genomics and Individualized Medicine Key Lab, Beijing Institute of Genomics, Chinese Academy of Sciences, Chaoyang District, Beijing, Peoples Republic of China; Penn State Hershey Cancer Institute, United States of America

## Abstract

Hypoxia inducible factor 1α (Hif1α) is a stress responsive transcription factor, which regulates the expression of genes required for adaption to hypoxia. Hif1α is normally hydroxylated by an oxygen-dependent prolylhydroxylase, leading to degradation and clearance of Hif1α from the cell. Under hypoxic conditions, the activity of the prolylhydroxylase is reduced and Hif1α accumulates. Hif1α is also constitutively expressed in tumor cells, where it is associated with resistance to ionizing radiation. Activation of the Hif1α transcriptional regulatory pathway may therefore function to protect normal cells from DNA damage caused by ionizing radiation. Here, we utilized the prolylhydroxylase inhibitor dimethyloxalylglycine (DMOG) to elevate Hif1α levels in mouse embryonic fibroblasts (MEFs) to determine if DMOG could function as a radioprotector. The results demonstrate that DMOG increased Hif1α protein levels and decreased the sensitivity of MEFs to ionizing radiation. Further, the ability of DMOG to function as a radioprotector required Hif1α, indicating a key role for Hif1α's transcriptional activity. DMOG also induced the Hif1α -dependent accumulation of several DNA damage response proteins, including CHD4 and MTA3 (sub-units of the NuRD deacetylase complex) and the Suv39h1 histone H3 methyltransferase. Depletion of Suv39h1, but not CHD4 or MTA3, reduced the ability of DMOG to protect cells from radiation damage, implicating increased histone H3 methylation in the radioprotection of cells. Finally, treatment of mice with DMOG prior to total body irradiation resulted in significant radioprotection of the mice, demonstrating the utility of DMOG and related prolylhydroxylase inhibitors to protect whole organisms from ionizing radiation. Activation of Hif1α through prolylhydroxylase inhibition therefore identifies a new pathway for the development of novel radiation protectors.

## Introduction

Hypoxia is associated with inflammatory diseases, tumors and wound healing [1]. Hypoxia increases the levels of the hypoxia inducible factor (HIF), a transcription factor that alters gene expression and promotes adaptation of cells to hypoxic environments. HIF is a heterodimeric transcription factor composed of the hypoxia inducible Hif1α subunit and the constitutively expressed Hif1β [2]. Under normal oxygen tension, Hif1α is hydroxylated by the HIF-prolylhydroxylase PHD2, facilitating interaction with the VHL E3 ubiquitin ligase complex [3], [4]. Hif1α is then targeted for ubiquitin-dependent degradation [5], and Hif1α protein levels remain low. Because HIF-prolylhydroxylases require oxygen to hydroxylate Hif1α [5], hypoxia inhibits PHD2 hydroxylase activity, decreasing hydroxylation of Hif1α, and allowing Hif1α to accumulate. Hif1α then forms an active transcriptional complex with Hif1β, and upregulates expression of more than 60 genes which are required for cells to survive under low oxygen tension, including angiogenic factors, erythropoietin, glycolytic enzymes and survival factors [6]. Activation of Hif1α therefore regulates multiple biological pathways which promote cell survival under conditions of stress [5], [7], [8].

Hif1α also plays a key role in both tumor growth and sensitivity of tumors to chemotherapy and radiotherapy. Many tumors express high levels of constitutive Hif1α which is associated with resistance to therapy and poor prognosis [9]–[12]. Reducing Hif1α expression using either genetic or pharmacological approaches both decreases tumor growth and sensitizes tumors to radiation therapy and to chemotherapy [13]–[16]. Further, cells in which Hif1α is inactivated, including both Hif1α^−/−^ MEFs [9] and tumor cell lines, exhibit increased sensitivity to both chemotherapy and radiotherapy [9], [14], [17], [18]. These observations clearly indicate that cells expressing high levels of Hif1α are more resistant to DNA damaging agents, presumably due to the transcriptional activation of pro-survival genes by Hif1α. Hif1α levels may also be increased by exposure to ionizing radiation [18], [19]. Although the contribution of Hif1α to mediating resistance to radiation therapy varies between cell and tissue types [16], [20], it is now clear that increased Hif1α levels can have profound impacts on the sensitivity of both normal and tumor cells to cancer therapy.

Several studies have identified inhibitors of the prolylhydroxylase which hydroxylates Hif1α and promotes its degradation [21]. These prolylhydroxylase inhibitors can be employed to stabilize Hif1α in cells so that the contribution of Hif1α to specific cellular pathways can be assessed. Prolyl hydroxylases (PH) are members of an extended family of Fe (II) and 2-oxoglutarate dependent dioxygenases [22], which function to hydroxylate proteins on multiple amino-acids, including proline [22], [23]. Small molecule inhibitors of prolylhydroxylases include compounds which are structural analogs to the 2-oxoglutarate co-factor required for these enzymes to function [22]. One of these, dimethyloxalylglycine (DMOG), is a cell-permeable inhibitor of both proline and asparaginyl hydroxylases [24], which can activate the Hif1α dependent gene expression both *in vitro* [25] and *in vivo* [26]. Prolylhydroxylase inhibitors such as DMOG have been shown to promote cell survival under conditions of hypoxia or growth factor deprivation by elevating levels of Hif1α [25], [27]–[29]. Elevating levels of Hif1α through inhibition of prolylhydroxylase function is therefore an effective method for modulating Hif1α function.

Currently, there is significant interest in the development of radioprotective agents which can be used to protect against exposure to ionizing radiation. This can include protection of normal tissue from radiation injury during radiation therapy of tumors, as well as developing medical countermeasures to protect large populations against accidental exposure to radiation. Elevated levels of Hif1α are strongly associated with radio-resistance [9]–[12], indicating that increasing Hif1α levels in normal tissues may function to protect them from radiation damage. Here, we demonstrate that activation of the Hif1α pathway by the prolylhydroxylase inhibitor DMOG can protect normal cells from exposure to radiation.

## Results

**Figure 1:**
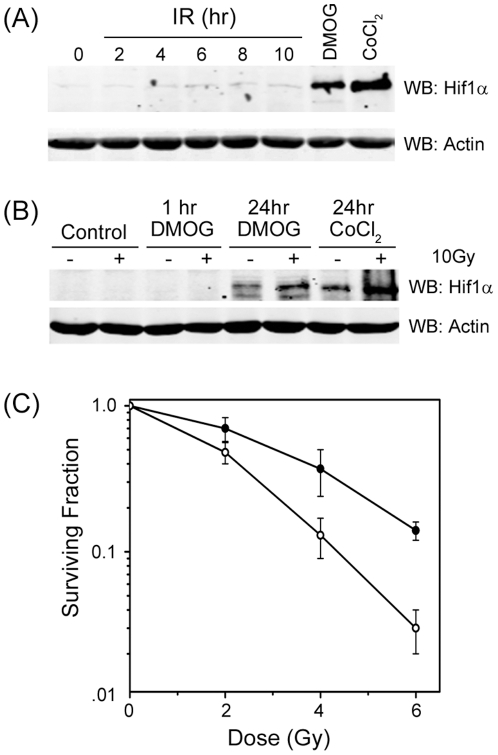
Upregulation of Hif1α promotes radioprotection. (**A**) MCF-7 cells were incubated in DMOG (1 mM) or CoCl_2_ (200 µM) for 10 hr or exposed to 10Gy of ionizing radiation (IR) for the indicated times. Cell extracts were prepared and examined by western blot (WB) analysis to monitor levels of Hif1α. β-actin levels are shown to demonstrate equal loading. (**B**) MCF-7 cells were incubated in DMOG (1 mM) or CoCl_2_ (200 µM) for the indicated times. Cells were irradiated (10Gy) at time zero where indicated. Cell extracts were prepared and examined by western blot (WB) analysis to monitor levels of Hif1α. β-actin levels are shown to demonstrate equal loading. (**C**) MEFs were preincubated in DMOG (1 mM) for 1 hr, followed by exposure to IR as indicated. 24 hours later, DMOG was removed and clonogenic cell survival assays carried out. • = DMOG, ○ = solvent (PBS).

To examine if activation of Hif1α impacted radiosensitivity, we first examined if exposure of MCF-7 cells to ionizing radiation (IR) lead to stabilization of the Hif1α transcription factor. MCF-7 cells were exposed to IR or treated with the prolylhydroxylase inhibitors DMOG [26] or CoCl_2_ [27]. Both DMOG and CoCl_2_ caused significant accumulation of Hif1α in MCF-7 cells (figure 1A), as previously reported [26], [27]. Exposure to IR alone had only a slight impact on accumulation of Hif1α, indicating that IR does not cause significant stabilization of Hif1α. However, combining IR with either DMOG or CoCl_2_ led to much larger accumulation of Hif1α protein than exposure to DMOG or CoCl_2_ alone (figure 1B). This indicates that DMOG and IR function synergistically to stabilize Hif1α and increase the accumulation of this protein in cells.

Previous work indicated that upregulation of Hif1α by treatment with the prolylhydroxylase inhibitor DMOG can improve survival under conditions of stress [25], [27]–[29]. Because DMOG functioned synergistically with IR to promote accumulation of Hif1α (figure 1B), we determined if the increased levels of Hif1α caused by DMOG affected radiosensitivity. For these experiments, MEFs were used. These were chosen because MEFs, unlike tumor derived or transformed cells, do not express basal levels of Hif1α which can impact intrinsic radiosensitivity [13]–[16]. This allows for the direct assessment of the impact of increased Hif1α expression on radiosensitivity. When MEFs were treated for 24 hr with DMOG to increase Hif1α levels, there was a significant decrease in radiosensitivity (figure 1C), indicating that DMOG functions as a radioprotector. Figure 1 therefore indicates that upregulation of Hif1α can protect MEFs from the cytotoxic effects of IR.

Hif1α functions as a transcriptional regulator [30], suggesting that Hif1α may control the transcription of DNA repair proteins (or apoptotic factors), which can protect cells from radiation damage. Recent work has implicated components of the NuRD deacetylase complex in the cells response to both hypoxia [31] and DSB repair [32], [33]. The NuRD complex, which contains both the CHD4 ATPase and the HDAC2 deacetylase, is required for cells to repair and survive IR-induced DNA damage [32], [33]. Importantly, expression of the MTA1 sub-unit of NuRD is induced under hypoxic conditions [31], [34], and both MTA1 and histone deacetylases contribute to Hif1α stability [34]. This indicates that components of the NuRD complex may be upregulated by Hif1α. Therefore, we examined if components of the NuRD complex were increased by hypoxia mimics and if this induction contributed to the observed radioprotection by DMOG.

**Figure 2:**
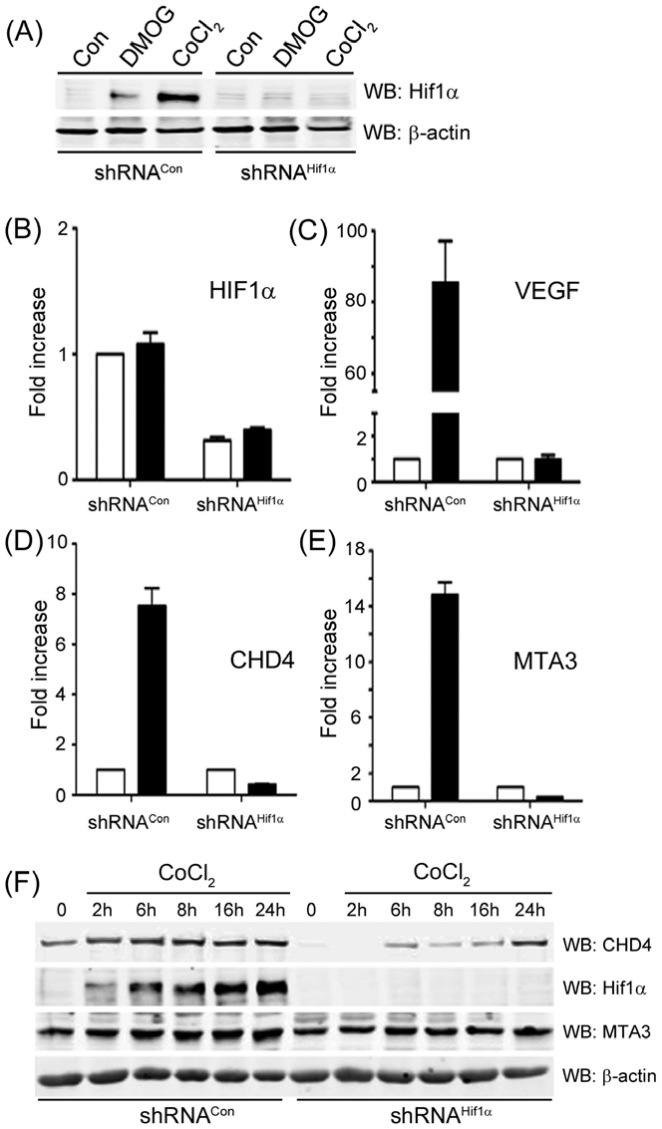
Hif1α increases transcription of the CHD4 and MTA3 genes. (**A**) MCF-7 cells stably expressing either a non-specific shRNA (shRNA^Con^) or shRNA targeting Hif1α (shRNA^Hif1α^) were exposed to DMOG (1 mM) or CoCl_2_ (200 µM) for 24 hrs. Cell extracts were prepared and then examined by western blot (WB) analysis to monitor levels of Hif1α. β-actin levels are shown to demonstrate equal loading. (**B–E**) MCF-7 cells stably expressing either a non-specific shRNA (shRNA^Con^) or shRNA targeting Hif1α (shRNA^Hif1α^) were exposed to solvent (open bars) or CoCl_2_ (200 µM; filled bars) for 8 hr. mRNA levels for the indicated genes were measured by RT-PCR as described in methods. Results ± SE (n = 3). (**F**) MCF7 cells stably expressing either a non-specific shRNA (shRNA^Con^) or shRNA targeting Hif1α (shRNA^Hif1α^) were exposed to CoCl_2_ (200 µM) for indicated number of hours. Cell extracts were prepared and then examined by western blot (WB) analysis to monitor levels of CHD4, MTA3 and Hif1α. β-actin levels are shown to demonstrate equal loading.

**Figure 3:**
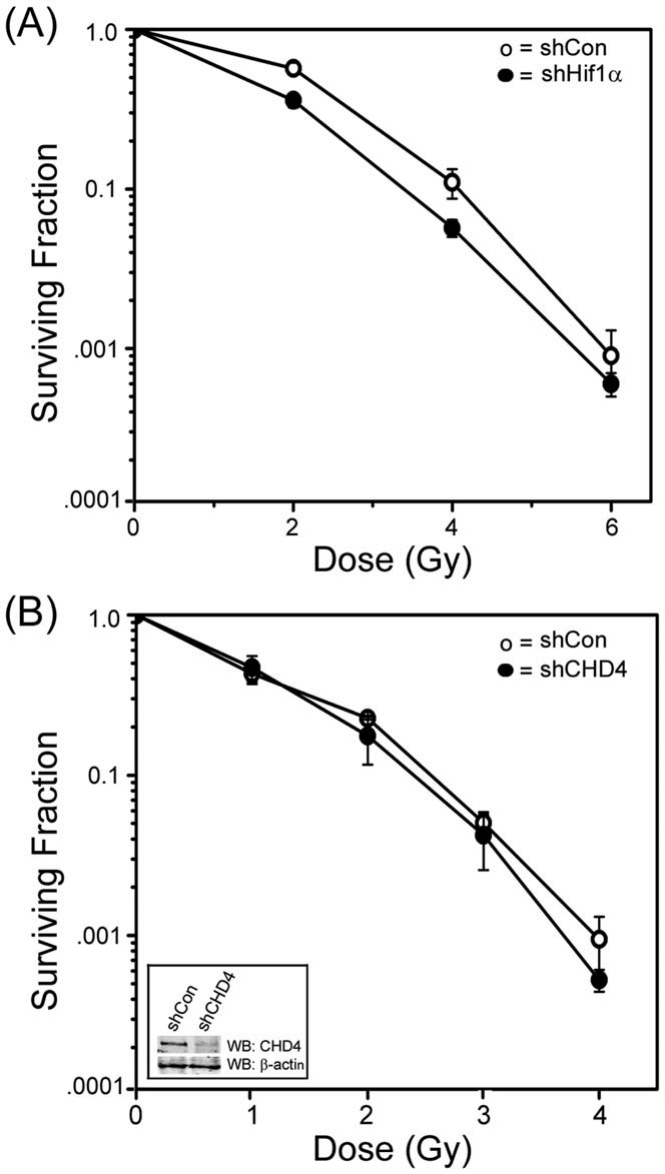
Radiosensitivity of cells lacking Hif1α or CHD4. (**A**) MCF-7 cells stably transfected with control (shCon) or Hif1α (sh^Hif1α^) shRNA or (**B**) HEK293T cells stably transfected with control (shCon) or CHD4 (shCHD4) shRNA were irradiated as indicated and clonogenic cell survival assays carried out. Western blot analysis of CHD4 protein levels shown in figure 3B (inset). Results ± SE (n = 3).

To examine the transcriptional activity of Hif1α, we first reduced expression of Hif1α using shRNA. shRNA targeting Hif1α blocked the accumulation of Hif1α after treatment with either DMOG or CoCl_2_ (figure 2A) and reduced the levels of Hif1α mRNA (figure 2B). Silencing of Hif1α also inhibited the accumulation of VEGF mRNA (figure 2C), a key target of Hif1α [30], demonstrating that Hif1α function is abolished in the shRNA^Hif1α^ cells. Next, we determined if Hif1α regulates expression of the CHD4 and MTA3 genes. Figure 2D and 2E demonstrate that activation of Hif1α by CoCl_2_ increased the levels of CHD4 and MTA3 mRNA, and this increase was abolished when Hif1α was silenced with shRNA (figure 2D and 2E). This demonstrates that Hif1α is required for the accumulation of CHD4 and MTA3 mRNA after exposure to CoCl_2_. Further, CoCl_2_ increased the levels of Hif1α, CHD4 and MTA3 protein with similar kinetics (figure 2F), consistent with the Hif1α-dependent increase in their mRNA levels (figure 2D and 2E). Significantly, suppression of Hif1α with shRNA also reduced the basal levels of CHD4 protein, whereas basal levels of MTA3 were unaffected by loss of Hif1α (figure 2E, 2F). Further, loss of Hif1α greatly attenuated the accumulation of CHD4 after exposure to CoCl_2_, but had only a small impact on the accumulation of MTA3 protein (figure 2F). Figure 2 therefore demonstrates that increased levels of Hif1α lead to increased levels of MTA3 and CHD4 mRNA, potentially identifying CHD4 and MTA3 as transcriptional targets for Hif1α. Significantly, loss of Hif1α decreased both the basal and stimulated levels of CHD4 protein, indicating that Hif1α plays a critical role in maintaining basal and stimulated levels of CHD4. Previous work indicates that CHD4 can participate in the cells response to IR-induced DNA damage [32], [33]. This suggests that, because cells expressing shRNA to Hif1α have decreased levels of CHD4, they should be more sensitive to IR. Figure 3A demonstrates that cells lacking Hif1α exhibit a small but significant increase in radiosensitivity, consistent with a key role for Hif1α in regulating radiosensitivity. However, when shRNA was used to deplete CHD4 protein levels to a level similar to those detected in Hif1α depleted cells (compare figure 2F and 3B, inset) no significant impact on radiosensitivity was seen (figure 3B). Similarly, silencing MTA3 expression with shRNA did not alter cellular radiosensitivity (data not shown). We interpret this to mean that, while Hif1α contributes to cell survival after exposure to IR (figure 1C and figure 3A), this regulation of radiosensitivity is not mediated through the ability of Hif1α to regulate the expression of either CHD4 or MTA3. Therefore, although we have identified CHD4 and MTA3 as potential transcriptional targets for Hif1α, the ability of Hif1α to protect cells from radiation damage does not require either CHD4 or MTA3. It is more likely that upregulating CHD4 and MTA3, which are components of NuRD deacetylase complex, plays a key role in other processes, such as transcriptional repression, which are a feature of the hypoxia response [4], [6], [30].

**Figure 4:**
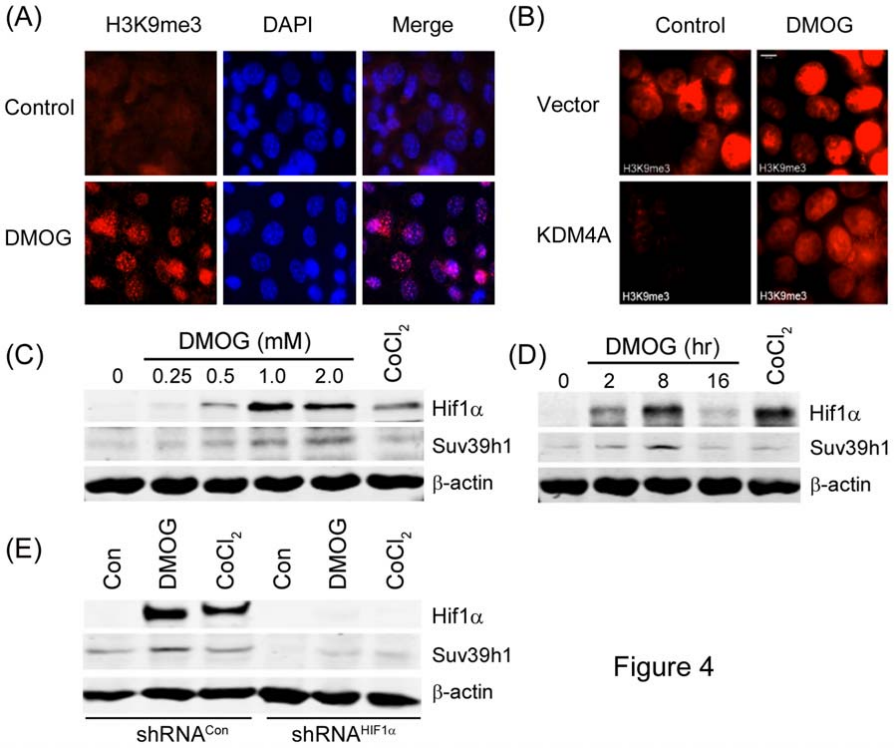
DMOG inhibits lysine demethylases and increases levels of the Suv39h1 methyltransferase. (**A**) MEFs were incubated in either solvent (Control: DMSO) or DMOG (1 mM) for 24 hr. H3K9me3 levels were detected by immunofluorescent staining with antibody to H3K9me3 and co-stained with DAPI to locate nuclear DNA. (**B**) HEK293T cells were transiently transfected with vector or KDM4A and allowed to recover for 30 hrs. Cells were either untreated or incubated with DMOG (1 mM) for 24 hrs. H3K9me3 levels were detected by immunofluorescent staining with antibody to H3K9me3. (**C**) MCF-7 cells were incubated in increasing concentrations of DMOG or CoCl_2_ (200 µM) for 24 hrs. Cell extracts were prepared and examined by western blot (WB) analysis to monitor levels of Hif1α and Suv39h1. β-actin levels are shown to demonstrate equal loading. (**D**) MCF-7 cells were incubated in DMOG (1 mM) or CoCl_2_ (200 µM) for the indicated time. Cell extracts were prepared and examined by western blot (WB) analysis to monitor levels of Hif1α and Suv39h1. β-actin levels are shown to demonstrate equal loading. (**E**) MCF-7 cells expressing either a non-specific shRNA (shRNA^Con^) or shRNA targeting Hif1α (shRNA^Hif1α^) were incubated with DMOG or CoCl_2_ (200 µM) for 24 hrs. Cell extracts were prepared and examined by western blot (WB) analysis to monitor levels of Hif1α and Suv39h1. β-actin levels are shown to demonstrate equal loading.

To further explore how stabilization of Hif1α regulates radiosensitivity, we examined a second group of proteins, the histone demethylases, which are transcriptionally activated by Hif1α. Histone methylation is a dynamic signal transduction process, controlled by histone methyltransferases [35], [36] and histone demethylases (KDMs) [37], [38]. Histone methylation plays a key role in regulating chromatin structure, transcriptional activity and the DNA damage response [39], [40]. The importance of histone methylation in the DNA damage response is highlighted by the observation that inactivation of H3K9me3 methyltransferases leads to genomic instability and an inability to correctly repair DSBs caused by IR [41], [42]. Several KDMs [43], including KDM4B and KDM3A [44], contain Hypoxia Response Elements (HRE) and are transcriptionally activated by Hif1α under hypoxic conditions [43], [44], suggesting that histone methylation may decrease under hypoxic conditions. Accordingly, we examined how the stabilization of Hif1α by DMOG impacts methylation of H3K9me3. In figure 4A, exposure of cells to DMOG rapidly increased H3K9me3 in cells. This was somewhat unexpected, since Hif1α transcriptionally upregulates 2 H3K9me3 specific KDMs, KDM4B and KDM3A [43], [44], and should therefore decrease H3K9me3 levels. However, KDMs and the Hif1α prolylhydroxylases belong to the larger family of dioxygenases [22]. Both the Hif1α prolylhydroxylases and KDMs utilize an Fe (II) and 2-oxoglutarate-dependent dioxygenase mechanism to either hydroxylate proline on Hif1α [4] or remove methyl groups from methylated lysines on histone tails [37], [38]. DMOG, which is a 2-oxoglutarate analog, is a competitive inhibitor of the Hif1α-PH and is therefore likely to inhibit KDMs as well. In figure 4B, the H3K9me3 specific demethylase KDM4A was transiently expressed in cells. Cells expressing vector showed increased H3K9me3 methylation when cells were exposed to DMOG, consistent with inhibition of endogenous KDMs. Overexpression of KDM4A resulted in almost complete loss of endogenous H3K9me3 in the cells; however, addition of DMOG to cells expressing KDM4A restored H3K9me3 levels to near normal. Figure 4B therefore clearly demonstrates that DMOG, in addition to increasing Hif1α protein levels, can also inhibit endogenous KDMs and therefore increase levels of H3K9me3 in the cells.

To further analyze how DMOG may regulate H3K9me3 levels, we also determined if Hif1α could increase expression of Suv39h1, a key H3K9me3 methyltransferase [42]. Surprisingly, figure 4C and 4D demonstrate that both DMOG and, to a lesser extent, CoCl_2_, increased expression of the Suv39h1 methyltransferase. Further, the increase in Suv39h1 protein closely followed the dose-dependent (figure 4C) and time-dependent (figure 4D) increase in Hif1α levels caused by DMOG. Importantly, both basal and DMOG dependent induction of Suv39h1 were abolished in cells lacking Hif1α (figure 4E), indicating that Hif1α directly regulates the levels of Suv39h1 in cells. Figure 4 therefore demonstrates that DMOG can influence H3K9me3 levels by directly inactivating H3K9 demethylases (figure 4A and 4B) as well as upregulating H3K9 methyltransferases through a Hif1α dependent mechanism Figure 4C–E).

**Figure 5:**
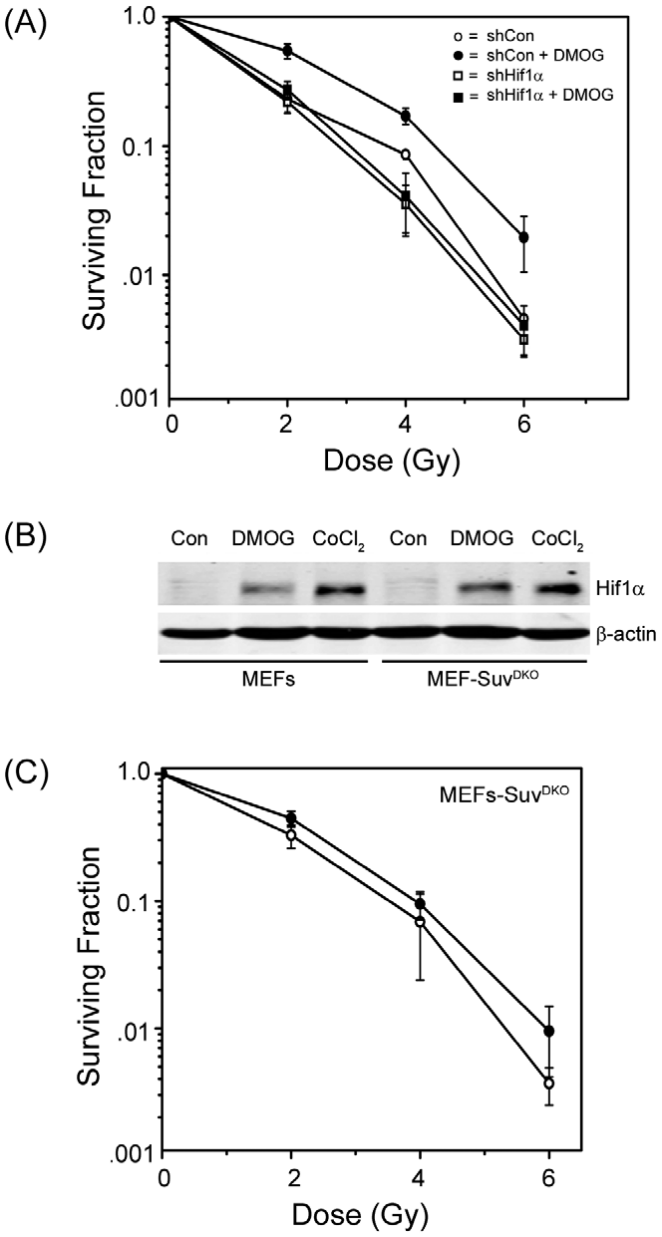
DMOG protects cells from radiation by stabilizing the Hif1α protein. (**A**) MEFs expressing a non-specific shRNA (○, •; sh^Con^) or shRNA targeting Hif1α (□, ▪; sh^Hif1α^) were untreated (○, □) or preincubated for 1 hr in DMOG (•, ▪; 1 mM), irradiated at the indicated dose and allowed to recover for 24 hr. DMOG was removed by medium exchange and clonogenic cell survival assays carried out following 10–12 days growth in culture. Results ± SE (n = 6). (**B**) Wild type MEFs or MEFs derived from mice with a double knockout of Suv39h1 and Suv39h2 (MEF-Suv^DKO^) were incubated with DMOG (1 mM) or CoCl_2_ (200 µM) for 24 hrs. Cell extracts were prepared and examined by western blot (WB) analysis to monitor levels of Hif1α. β-actin levels are shown to demonstrate equal loading. (**C**) MEFs derived from mice with a double knockout of Suv39h1 and Suv39h2 (MEF-Suv^DKO^) were incubated with solvent (○) or DMOG (•; 1 mM) for 1 hr, irradiated at the indicated dose and allowed to recover for 24 hrs. DMOG was removed by medium exchange, cells allowed to recover for 10–12 days, and clonogenic cell survival assays carried out. Results ± SE (n = 3).

The previous results indicate that DMOG may influence radiosensitivity through 2 distinct pathways. First, DMOG can directly inhibit KDMs, increasing H3K9me3 levels by a mechanism which is independent of Hif1α. Second, DMOG can directly inhibit Hif1α-prolylhydroxylases, leading to stabilization and accumulation of transcriptionally active Hif1α. To determine the relative contributions of these 2 pathways to the ability of DMOG to function as a radioprotector, we examined how silencing of Hif1α with shRNA impacted radioprotection. Figure 5A demonstrates that DMOG increased radioresistance in MEFs expressing a non-specific shRNA, but this effect was lost when Hif1α was silenced. The ability of DMOG to function as a radioprotector therefore requires Hif1α, indicating that DMOG exerts its primary effect on radiosensitivity through inhibition of the Hif1α-prolylhydroxylase and accumulation of Hif1α. Therefore, although DMOG can also inhibit KDMs (figure 4A and 4B), this inhibition does not appear to be critical for the observed radioprotection.

Finally, we examined if the Hif1α -dependent increase in expression of the Suv39h1 methyltransferase was required for DMOG dependent radioprotection. For this, we determined if DMOG can protect MEFs lacking both the Suv39h1 and Suv39h2 methyltransferases (Suv^DKO^ cells; [42]). The ability of DMOG and CoCl_2_ to increase Hif1α levels was not altered in the Suv^DKO^ MEFs (figure 5B). Further, whereas DMOG functioned as a radioprotector in normal MEFs (figure 5A), the protective effect of DMOG was significantly reduced in the Suv^DKO^ MEFs (figure 5C). This indicates that the reduced ability of DMOG to protect Suv^DKO^ MEFs may be attributed to both the loss of Hif1α dependent increase in the Suv39h1 methyltransferase, as well as changes in other Hif1α target genes. Overall, figure 5 demonstrates that DMOG can protect cells form radiation by stabilizing the Hif1α transcription factor, and increasing expression of genes, including the Suv39h1 methyltransferase, which can promote cell survival.

**Figure 6:**
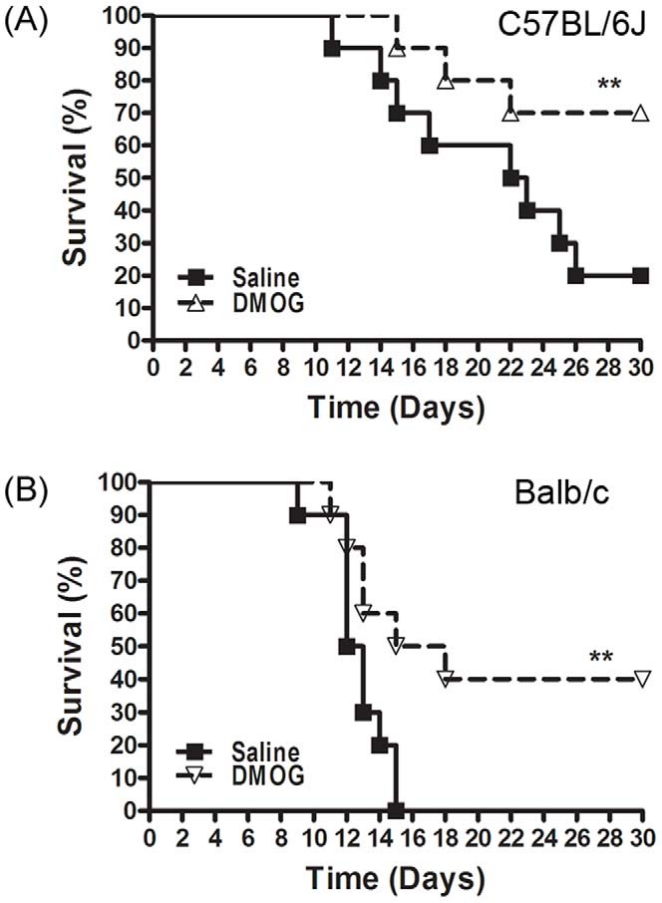
DMOG improves survival of mice following total body irradiation. Mice were irradiated (8Gy TBI) and survival monitored over 30 days. DMOG (100 mg/kg) or saline was administered ip 4 hrs before TBI, and 12 hrs and 36 hrs after TBI. Kaplan-Meier survival plots of (**A**) C57BL/6J (**p = 0.037) and (**B**) Balb/c mice (**p = 0.019) are shown.

Finally, we sought to determine if DMOG could protect cells from DNA damage at the level of the whole organism. Hif1α activation can transcriptionally activate a wide range of genes, including VEGF, which promote angiogenesis, and erythropoietin, which mobilizes bone marrow and improves blood parameters [29], [45]. It is likely that these factors may be important in promoting recovery of sensitive tissues, such as the bone marrow and gastrointestinal tract, from total body irradiation [46]. In order to assess if the radioprotective effects of DMOG can be detected at the level of the whole organism, we examined if DMOG had protective effects in a murine total body irradiation (TBI) model. DMOG was injected ip into either C57BL/6J (figure 6A) or Balb/c mice (figure 6B), two widely used murine TBI model systems. DMOG alone did not cause any toxicity, with 100% of the mice surviving at 30 days (data not shown), in agreement with previous studies using DMOG [47]. TBI at 8Gy caused 80% and 100% lethality in saline-treated C57BL/6J and Balb/c mice respectively (figure 6A and 6B). However, DMOG treatment significantly improved the survival of both C57BL/6J and Balb/c mice and rescued them from radiation induced lethality. Activation of Hif1α by DMOG results in the transcriptional upregulation of many genes and growth factors and is associated with increased cell survival in culture and protection of mice from whole body irradiation. These results clearly demonstrated that DMOG exhibits radioprotective potential in murine models.

## Discussion

The Hif1α transcriptional regulatory protein controls the expression of genes which promote cell survival under conditions of low oxygen tension [2]. Hif1α can switch cell metabolism towards increased expression of growth factors and anti-apoptotic factors as well as switching of metabolic pathways, allow the cell to maintain energy levels under hypoxic conditions [2], [4], [6]. Hif1α levels are controlled through direct hydroxylation of Hif1α by the PHD2 prolylhydroxylase, leading to degradation of Hif1α under normal oxygen tension [3]–[5]. Small molecule inhibitors of prolylhydroxylases have been developed which inhibit PHD2 and related enzymes, leading to stabilization of Hif1α and upregulation of the hypoxia response [8], [25], [48]. Here, we have shown that stabilization of Hif1α using the prolylhydroxylase inhibitor DMOG protects both MEFs and mice from the cytotoxic effects of exposure to IR. This is consistent with previous studies demonstrating that tumor cells containing constitutively high levels of Hif1α are more resistant to both chemotherapy and radiotherapy [9]–[16]. Increasing Hif1α levels in normal cells with prolylhydroxylase inhibitors such as DMOG therefore represents a novel pathway for the development of new and effective radioprotective agents.

Our results clearly show that DMOG requires Hif1α to protect cells from radiation, since suppression of Hif1α with shRNA abolished the protective effect of DMOG. A key question is to determine how stabilization of Hif1α by DMOG can promote radioprotection. Because Hif1α functions as a transcriptional activator, it likely exerts its protective effects through increased expression of genes involved in DNA repair or cell survival. Previous studies indicate that hypoxia can alter expression of components of the mismatch repair pathway [49], [50]; however, altered expression of these proteins would not account for radioprotection observed after activation of Hif1α by DMOG. However, we identified 3 new targets for Hif1α - the CHD4 helicase, the MTA3 regulatory protein and the Suv39h1 methyltransferase. All 3 were increased at both the mRNA and protein level by DMOG and the hypoxia mimetic agent CoCl_2_. Further, this increase in mRNA required the Hif1α transcription factor, suggesting that Hif1α can directly regulate their transcription. Previous work has shown that MTA1, a close family member related to MTA3, is also transcriptionally upregulated by Hif1α during hypoxia [31], implying that the MTA family of coactivators are a common target for the Hif1α transcription factor. Both CHD4 and MTA3 are components of the NuRD complex, a histone deacetylase complex which is implicated in DNA repair [32], [33]. Inactivation of CHD4 leads to increased sensitivity to IR-induced DNA damage [32], [33], indicating a key role in the repair of DSBs. Taken together, this would suggest that increased levels of NuRD may protect cells from radiation. However, although Hif1α was important for DMOG to protect cells from IR, loss of either CHD4 or MTA3 expression did not alter sensitivity to IR in our cell system. We interpret this to mean that, although DMOG can stabilize Hif1α and increase levels of the NuRD complex, the accumulation of NuRD does not significantly impact the radiosensitivity of the cells. The accumulation of the NuRD deacetylase complex may therefore play an alternate role in the hypoxia response, such as transcriptional repression of genes during low oxygen tension.

An alternate explanation for how DMOG may protect cells from radiation damage can be proposed based on the previous observation that Hif1α can alter expression of genes involved in the regulation of histone methylation. Hif1α increases expression of histone demethylases, including KDM4A and KDM4B [43], [44], which function to remove methyl groups from methylated histones on the chromatin. The increased expression of KDMs during hypoxia is associated with a decrease in histone methylation, including a reduction on H3K9me3 levels [43], [44]. Previous work has shown that a decrease in H3K9me3 levels is associated with an increase in radiosensitivity [41]. Hif1α -dependent increases in KDM expression would therefore be predicted to increase, rather than decrease, radiosensitivity. However, we found that, unlike hypoxia [43], [44], DMOG increased rather than decreased H3K9me3 levels. This result is explained by the fact that KDMs and Hif1α-prolylhydroxylases share a common catalytic mechanism [22], so that both classes of enzyme are inhibited by DMOG (figure 4B). DMOG therefore inhibits the Hif1α-prolylhydroxylase, leading to accumulation of transcriptionally active Hif1α and upregulation of KDMs [43], [44]. However, because DMOG can also directly inhibit KDMs (figure 4b), this essentially negates the increased expression of KDMs mediated by Hif1α. Overall, DMOG will increase histone methylation through inhibition of KDMs. Further, cells in which Hif1α was inactivated were not protected by DMOG, despite the ability of DMOG to increase H3K9me3 levels in these cells. We conclude that the ability of DMOG to protect cells from IR is mediated through the transcriptional activity of Hif1α rather than through a general inhibition of endogenous KDMs in the cell.

In addition to CHD4 and MTA3, we also identified the Suv39h1 methyltransferase as a target of the Hif1α. Suv39h1 is a key player in the di- and trimethylation of H3 on lysine 9 [42]. Further, loss of Suv39h1 and decreased levels of H3K9me3 are associated with increased radiosensitivity [41] and decreased genomic stability [42]. Secondly, DMOG can also increase the expression of Suv39h1 through activation of Hif1α, a process which will also tend to increase H3K9me3 levels. Importantly, the ability of DMOG to function as a radioprotector was significantly reduced (but not abolished) in MEFs which lacked expression of Suv39h1 and Suv39h2. Since DMOG increases Suv39h1 expression through a Hif1α dependent mechanism, this indicates that the main contributor to DMOG mediated radioresistance is the transcriptional upregulation of the Suv39h1 methyltransferase by Hif1α. How can increased expression of Suv39h1 impact radiosensitivity? As discussed above, cells lacking Suv39h1 have significant defects in both H3K9me3 and in DSB repair [41], [42]. DMOG may therefore increase expression of Suv39h1, leading to increased methylation of H3K9 and allowing for more efficient activation of the DNA damage response. Suv39h1 may therefore methylate H3K9 within specific regions of the chromatin after DNA damage in order to improve the efficiency of repair. However, it is also possible that the radioprotective effects of increased Suv39h1 are not directly on the DNA repair machinery, but instead feedback through altered methylation of key genes, such as anti-apoptotic proteins. Overall, the protective effect of DMOG is largely mediated through the Hif1α dependent increase in expression of the Suv39h1 methyltransferase, leading to increased H3K9me3 levels in the cell.

Finally, we also demonstrated that when DMOG was given to mice prior to irradiation it can protect them from total body irradiation. Significant improvement in survival was found in 2 different mouse strains, underlining the effectiveness of DMOG in a whole animal model. Previous studies using DMOG and related prolylhydroxylase inhibitors in whole animal models have indicated protection from ischemic injury [51], protection in a murine model of colitis [47] and the development of hypoxia tolerance [29]. A key target of Hif1α are the growth factors VEGF and erythropoietin [2]. DMOG can detectably increase erythropoietin levels in animal models [29], [45], leading to improvement in blood parameters, and can act to increase angiogenesis and muscle recovery from ischemic injury [26]. The most sensitive tissues to IR are the GI tract and bone marrow [46]. The ability of DMOG to stimulate the production of factors such as erythropoietin and VEGF, which can stimulate repopulation of the hematopoietic progenitors and promote formation of new vasculature, are likely to be critical factors in the ability of DMOG to protect whole animals from radiation.

Effective radioprotectors and radiation-mitigating agents are needed in the clinic to treat the radiation victims and to protect individuals from radiation exposure resulting from nuclear disasters or radiological attack. Many small molecules, including anti-oxidants, cytokines, activators of NF-KappaB and cyclin-dependent kinase inhibitors have been shown to have radioprotective effects in murine TBI models [52]–[57]. Our studies demonstrate that the prolyl hydroxylase inhibitor DMOG is an effective radioprotector in both tissue culture and whole animal models. Activation of Hif1α evokes a complex response at both the cellular and whole organism levels. Changes in gene transcription caused by DMOG at the cellular level, including changes in histone methylation, can impact the ability of individual cells to repair and survive radiation exposure. In addition, the ability of DMOG to stabilize Hif1α and stimulate production of growth factors such as VEGF and erythropoietin can promote DNA repair, the repopulation of sensitive cell types and promote survival at both the level of individual tissues and the whole organism. Manipulation of the Hif1α transcriptional pathway may reveal new targets for the development of novel radioprotective agents.

## Materials and Methods

### Cell Culture

HEK293T, MCF-7 (ATCC, NJ) and MEF [42] cells were cultured as previously described [42], [58]. Cells were irradiated using a Cs^137^ irradiator and clonogenic cell survival monitored as in [58]. Dimethyloxalylglycine was purchased from Frontier Scientific (Logan, UT). HEK293T, MCF7, and MEF cells were transfected using Lipofectamine 2000 according to the manufacturer's instructions (Invitrogen, CA), and selected using puromycin. Lentiviral Hif1α shRNA and GFP control shRNA were obtained from The RNAi Consortium (Broad Institute, Cambridge, MA). Plasmids were packaged with VSV-G expressing constructs phCMV-G and PCMVδ8.2 plasmid and transfected into HEK293T cells using Lipofectamine 2000. After 48 h, viral particles were harvested from the culture medium and used to prepare stable cell lines expressing GFP or Hif1α shRNA after selection with puromycin. The histone demethylase KDM4A was transiently expressed in HEK293T cells using Fugene-6 (Roche, IN) for 30 hr prior to use for experimental protocols.

### Cell lysates and Western blot analysis

Antibodies used: m Hif1α (Santa Cruz Biotechnology, CA); hHIF1α (Abcam, CA); H3K9me3 (Millipore, NY); β-actin (Cell Signaling Technology, MA); CHD4 and MTA3 (Bethyl Laboratories, TX); Suv39h1 (Novus, CA). To prepare nuclear lysates, cells were resuspended in 500 µl buffer A (10 mM Tris, pH8.0; 1 mM EDTA, pH8.0; 150 mM NaCl; 0.5% NP-40; 1 mM PMSF; 5 µg/ml leupeptin; 20 µg/ml aprotinin), incubated on ice for 15 min and centrifuged at 500 g for 5 min at 4°C. Supernatant was removed and the pellet resuspended in buffer B (20 mM Hepes, pH7.9; 400 mM NaCl; 1 mM EDTA; 0.5 mM DTT; 1 mM PMSF; 5 µg/ml leupeptin; 20 µg/ml aprotinin), incubated on ice for 15 min and cleared by centrifugation (21,000 g for 5 min at 4°C). For western blots, equal amounts of protein (Bradford Protein Assay kit, Bio-Rad Laboratories, CA) were separated by electrophoresis and transferred to nitrocellulose membranes (Bio-Rad Laboratories, CA). Membranes were blocked with Odyssey Blocking Buffer (Li-Cor, NB), incubated with primary antibodies for 3 h, and washed in PBST (PBS and 0.1% Tween-20) followed by goat anti–mouse IR Dye-800CW or goat anti-rabbit IR Dye-680CW secondary antibodies (Li-Cor, NB). Imaging was performed using the Li-Cor Odyssey Near Infra Red System, and analyzed using the Odyssey software system 3.0 package (Li-Cor, NB).

### RT-PCR

Total RNA was prepared using RNeasy kit (Qiagen, CA). 1 µg RNA and supplied random primers were used to generate cDNA with aid of QuantiTect reverse transcription kit (Qiagen, CA). RT-qPCR was performed using following primers: for Hif1α 5′-AACATAAAGTCTGCAACATGGAAG and 5′-TTTGATGGGTGAGGAATGGG; for CHD4 5′-CAGAGCTATTGGAATCACAGGG and 5′-TCGCTCATACTTCACTGTTGG; for MTA3 5′-GAGGCTGACTTGACCGATAAG and 5′-TGTCTCATTCAGAAGGGCAAC; VEGF primers were used as positive control and 18S RNA primers were used as internal control.

### Immunofluorescence

Cells (on cover slides) were fixed in phosphate-buffered saline (PBS) containing 2% paraformaldehyde. Cells were permeabilized in 0.2% Triton X-100 in PBS for 5 mins, and then blocked in fetal bovine serum for 20 min. Primary antibodies were prepared in 10% fetal bovine serum supplemented with 0.2% saponin. After a 1 h incubation with primary antibody, cells were washed three times with 0.2% Tween-20 and incubated for 1 h in secondary antibody (conjugated to either Texas Red or FITC; Santa Cruz Biotechnology, CA). Slides were mounted with Fluoromount-G (Southern Biotech, AL). Images were collected with an AxioImager Z1 microscope (Carl Zeiss, Inc.) equipped with a color digital camera (Axiocam MRc Rev.3; Carl Zeiss, Inc.) and Plan Apochromat oil M27 lens (63×, NA 1.4). Acquisition software and image processing used the AxioVision software package (Carl Zeiss, Inc.).

### Mice

This study was carried out in strict accordance with the recommendations in the Guide for the Care and Use of Laboratory Animals of the National Institutes of Health. The protocol was reviewed and approved by the Dana-Farber Cancer Institute's Animal Care and Use Committee (Protocol number 06-029). All efforts were made to minimize suffering. Male Balb/c mice were purchased from Taconic (NY, USA) and C57BL/6J mice were purchased from The Jackson Laboratory (ME, USA). For total body irradiation (TBI), mice were irradiated at the dose rate of 110 cGy/min using a 137Cs source (Gammacell® 40Exactor, Best Theratronics, Ottawa, Canada). DMOG (dissolved in saline) or saline was administered IP before and after TBI. The mice were given normal water that had undergone reverse osmosis and powdered food (Purina Pico Lab Chow) after TBI. The animals were closely inspected after TBI for ill-health and the moribund animals were sacrificed according to the guidelines in our approved ACUC protocol. The Kaplan-Meier survival curves were plotted for saline-treated and DMOG-treated cohorts. The statistical analysis of the Kaplan-Meier survival curves was performed with Graph Pad Prism Version 5 using Mantel Log-rank test [59].
